# Diagnostic and Clinical Significance of Serum Levels of D-Lactate and Diamine Oxidase in Patients with Crohn's Disease

**DOI:** 10.1155/2019/8536952

**Published:** 2019-08-19

**Authors:** Jierui Cai, Hong Chen, Meiling Weng, Shuyu Jiang, Jie Gao

**Affiliations:** ^1^School of Medicine, Southeast University, Nanjing, China; ^2^Department of Gastroenterology, Zhongda Hospital of Southeast University, Nanjing, China

## Abstract

**Background:**

Crohn's disease (CD) is a chronic intestinal inflammatory disease. An ideal laboratory marker that can predict the prognosis in terms of relapse of the disease is clinically desirable.

**Methods:**

A total of 59 CD patients were enrolled in this study. Enzyme-Linked Immunosorbent Assay (ELISA) was used to quantitatively detect the content of D-lactate (D-LA) and the diamine oxidase (DAO) levels in sera obtained from patients and 28 healthy controls. The correlation between these two biomarkers and disease activity scores was assessed. In addition, the ROC curve was used to evaluate the diagnostic accuracy of these two biomarkers.

**Results:**

The levels of D-LA in the serum of CD patients in the active stage and remission stage were 16.08 ± 4.8 mg/L and 11.16 ± 3.17 mg/L, respectively, and the difference was statistically significant (*t* = 4.67, *P* < 0.001). DAO levels were significantly higher in patients with the active stage compared to controls. The levels of D-LA and DAO in CD patients were positively correlated with the disease activity (*r* = 0.68 and 0.53, respectively, *P* < 0.05). The area under the ROC curve (AUC) when CD activity was diagnosed with D-LA and DAO alone was 0.815 and 0.748, respectively. The diagnostic efficacy of the two biomarkers was not significantly different from that of the erythrocyte sedimentation (ESR) and hypersensitive C-reactive protein (CRP) (*P* > 0.05). However, the area under the curve was 0.861 (0.746, 0.937) when the diagnosis was performed using a combination of D-LA, DAO, CRP, and ESR, which was significantly higher than when CRP or ESR were tested alone (*P* < 0.05).

**Conclusions:**

D-LA and DAO have a good prognostic value for CD activity. Rational combined use of biomarkers can significantly improve the diagnostic efficiency.

## 1. Introduction

Crohn's disease (CD) is a chronic intestinal inflammatory disease whose etiology remains unknown. Currently, there is no cure for Crohn's disease. The goal of medical treatment is to control and maintain the intestinal inflammation within the range of a remission stage or prevent deterioration of the healing state of intestinal mucosa. The endoscopy examination with histology is mostly used to make a diagnosis, to assess the severity of disease, and to evaluate the recovery of intestinal mucosa after treatment. However, endoscopic procedure is costly, invasive, and time-consuming and it is associated with the risk of multiple complications.

An ideal laboratory marker that can detect disease activity and monitor the effectiveness of treatment, and also provide a prognostic value for relapse of the disease, is clinically desirable. Two biomarkers have been extensively studied in CD: C-reactive protein (CRP) and erythrocyte sedimentation rate (ESR). It has been shown that these two markers correlate significantly with endoscopic lesions and risk of relapse and with the response to therapy [1]. However, the sensitivity and specificity of these two markers are far from being satisfactory [2]. When CD patients suffer from a systemic infection or autoimmune connective tissue diseases, the two markers are not sufficiently accurate to detect the activity of CD, which leads to late treatment due to the inability to make a timely diagnosis.

Development of a set of new monitoring biomarkers that are more sensitive and specific is clinically important. Increasing evidence indicates that damage to the intestinal barrier increases epithelial permeability which triggers inflammatory processes and is closely related to CD activity [3]. D-lactate (D-LA) is a product released by many of microflora residing in the human gastrointestinal tract. Thus, a rise in blood D-LA level reflects an increase in the permeability of intestinal mucosa [4]. In other words, the levels of circulating D-LA may reveal the degree of damage to the intestinal mucosa. Diamine oxidase (DAO) is synthesized only in the epithelial cells of intestinal villi. Damaged mucosal cells release DAO which increases its serum concentration. Given that activity of DAO is stable in the blood [5], the concentration of DAO in the blood may reflect the damage and restoration of the intestinal cavity in a timely manner. High concentration of D-LA and DAO in the blood is tightly linked to abnormal intestinal barrier function [6], implying that they can be used as sensitive and accurate markers for monitoring CD activity. A study has already observed that serum levels of D-LA and DAO in CD patients are markedly higher than those in normal control group and they decrease after treatment [7].

The present study was designed to assess the relationship between the concentration of D-LA, DAO, and Crohn's disease activity. It is also aimed at determining whether circulating D-LA and DAO can be used as promising molecular indicators of the activity of Crohn's disease.

## 2. Methods

### 2.1. Subjects

A total of 59 CD patients who were admitted at the Zhongda Hospital affiliated to Southeast University from October 2017 to April 2018 were included in the study. The CD diagnostic criteria were based on the *Consensus Opinions on the Diagnostic and Treatment Specifications for Inflammatory Bowel Diseases in China*. The patients were categorized by disease location (L1 ileum, L2 colon, and L3 ileocolon) according to the Montreal classification [8]. The evaluation of disease activity was carried out according to Crohn's Disease Activity Index [9]. In addition, 28 healthy individuals from the physical examination center were taken as the control group. The study was approved by the Research Ethics Committee of the Zhongda Hospital affiliated to Southeast University (Nanjing, China).

### 2.2. Research Method

This is a cross-sectional study. A total of 59 CD patients and 28 healthy people participated in this study. After clinical evaluations, their blood samples were obtained. The concentration of D-LA and DAO in the serum of the subjects was determined using ELISA. The ELISA kit was purchased from Beijing Zhongsheng Jinyu Diagnostic Technology Company. The concentration of ESR and CRP in the blood of patients was determined using routine testing methods. Moreover, other clinical data including loose stool times, abdominal pain degree, general situation, extraintestinal manifestations, drug use, abdominal mass, and hematocrit were weighted and collected, and the CDAI score of each patient was calculated. The remission stage was defined as CDAI < 150 points, and the active disease stage was defined as CDAI ≥ 150 points.

### 2.3. Statistical Analysis

Statistical analysis was performed using SPSS 23.0 and MedCalc 15.8 software. The measurement data conforming to the normal distribution is presented as *x* ± *s*, and the *t*-test was used for comparison between two groups of independent sample. Analysis of variance was used to compare multiple groups of data. The Pearson correlation coefficient was used to assess the correlation between the two variables. The ROC curve was used to evaluate the diagnostic efficacy, and the areas under the ROC curve were compared using *Z*-test. The group differences were considered statistically significant at the 5% level (*P* < 0.05).

## 3. Results

### 3.1. Characteristics of Study Subjects

A total of 59 patients were included in the study. Among them, 29 were in the active stage and 30 were in the remission stage. Patients who were in the active stage aged between 20 and 47 years old, with an average age of 37.2 ± 8.3 years old (including 13 males and 16 females). Patients in the remission stage aged between 17 and 55 years old, with an average age of 39.6 ± 13.3 years old (including 17 males and 13 females). The average age of the control group was 43.6 ± 13.8 years old (including 13 males and 15 females). There was no significant difference in sex, age, and other basic characteristics among the groups (*P* > 0.05) (Table 1).

### 3.2. Analysis of Levels of D-LA and DAO

The serum levels of D-LA in CD patients who were in the active and remission stages were 16.08 ± 4.8 mg/L and 11.16 ± 3.17 mg/L, respectively, and the difference between the two groups was statistically significant (*t* = 4.67, *P* < 0.001) (Table 2). There was no significant difference in serum D-LA levels between the remission stage CD patients and the healthy controls (10.2 ± 3.22 mg/L) (*t* = 1.13, *P* > 0.05). The serum levels of DAO in CD patients who were in the active and remission stages were 11.01 ± 4.49 U/L and 7.7 ± 3.44 U/L, respectively, and the difference between the two groups was statistically significant (*t* = 3.18, *P* < 0.05) (Table 2). However, there was no significant difference in serum DAO levels between the remission stage CD patients and the healthy controls (6.21 ± 2.34 U/L) (*t* = 1.91, *P* > 0.05).

### 3.3. Correlation between D-LA, DAO, and CD Disease Activity

**Figure 1:**
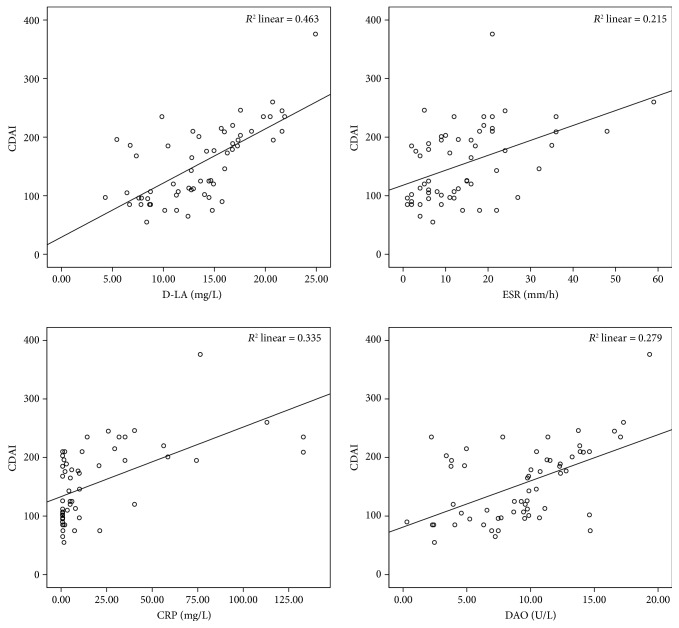
Correlation analysis of D-LA, DAO, ESR, CRP, and CDAI score.

According to the Pearson correlation analysis, the serum level of D-LA in CD patients was correlated with disease activity scores (*r* = 0.68, *P* < 0.05). The serum level of DAO in CD patients was also correlated with the disease activity scores (*r* = 0.53, *P* < 0.05). Moreover, CRP and ESR were correlated with the disease activity scores (*r* = 0.58 and 0.46, respectively, *P* < 0.05) (Figure 1).

### 3.4. Accuracy and ROC Analyses of D-LA, DAO, ESR, and CRP

**Figure 2:**
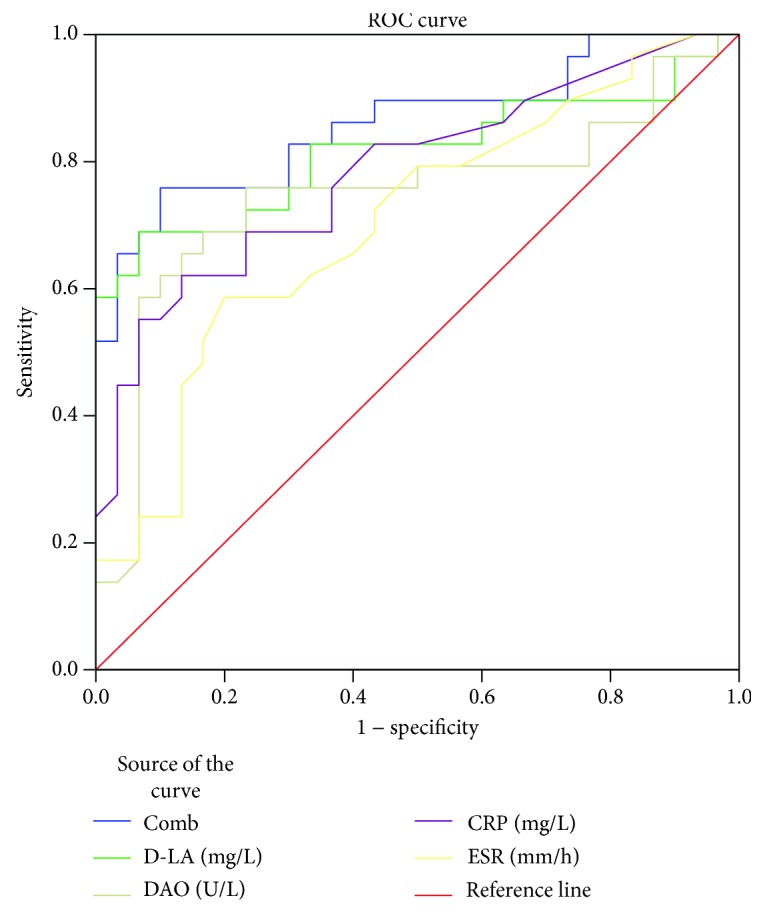
Receiver operating characteristic curves of the various biomarkers in CD active compared to CD remission.

According to the ROC curve, it was observed that serum D-LA and DAO have prognostic values for CD activity (*P* < 0.05). This was the case for the traditional biomarkers: ESR and CRP. The Youden index of each biomarker when they were used to diagnose CD activity was calculated. The maximum value reflected the evaluation accuracy. At 14.91 mg/L, the sensitivity and specificity of D-LA in diagnosing disease activity were 69% and 93%, respectively. At 9.75 U/L, the sensitivity and specificity of DAO in diagnosing disease activity were 76% and 77%, respectively. The area under ROC curve of the four biomarkers of CD activity was D-LA, CRP, DAO, and ESR in a descending order. All areas under ROC curve were compared with *Z*-test. There were no statistically significant differences between the groups (*P* > 0.05). Also, we applied the logistic regression model to generate combined predictors (Comb) as the analysis index. When D-LA, DAO, CRP, and ESR were used in combination with diagnosis, the corresponding area under the ROC curve was 0.861 (0.746, 0.937), which was significantly higher than ESR and CRP when they were used alone to evaluate CD activity (*P* < 0.05). Therefore, combining predictors relatively improved the diagnostic sensitivity and specificity (Table 3 and Figure 2).

## 4. Discussion

CD is a chronic intestinal inflammation and its pathogenesis remains unclear. Accumulating evidence suggests that disruption of the intestinal barrier function is associated with the occurrence and development of CD [10]. The mechanical barrier is the most important part of the intestinal mucosal barrier, and it is the histological foundation that maintains intestinal barrier function. It prevents the entry of various harmful substances and pathogen into the intestine [11]. The integrity of the mechanical barrier is regulated by the tight junctions between intestinal epithelial cells. The tight junctions are mainly composed of various intracellular proteins such as occlusive protein and filamentous actin. Studies have shown that the expression of these proteins in the junctional complex of colonic mucosa in CD patients is downregulated compared with that in normal people [12], which weakens the mechanical barrier and in turn induces inflammatory bowel disease. Studies have also shown that intestinal crypt epithelial cell apoptosis is positively correlated with the degree of colonic inflammation [13]. Microbial barriers are mainly associated with the intestinal flora. Recent advances in the use of genetic detection tools have increased our understanding on the role of the intestinal flora in CD. The microbial barrier formed by the intestinal flora can suppress CD progression. It has been reported in some studies that the use of probiotics or certain bacterial precursors can improve CD [14]. Kruis et al. suggested that nonpathogenic *Escherichia coli Nissle 1917* induced remission of intestinal inflammation and displayed high curative efficacy equivalent to that of mesalazine [15]. Butyrate and short-chain fatty acids are products of anaerobic fermentation in the bowel. They reduce intestinal PH levels and provide energy for colonic epithelial cells. However, the bacterial composition of the intestines of patients with CD is altered, resulting in lower levels of butyrate and short-chain fatty acids. This impairs the metabolism of intestinal epithelial cells, which in turn damages epithelial cells and ultimately induces intestinal inflammation [16]. The studies mentioned above have shown that destruction of the microbial barrier is closely related to the occurrence of CD.

Some prospective studies have suggested that intestinal mucosal permeability can be used as an indicator of inflammatory bowel disease. Tibble et al. demonstrated, through a prospective clinical study on 43 CD patients, that the increase in intestinal mucosal permeability of patients with active CD will increase the probability of complications and recurrence [17]. In recent years, many biomarkers have been used to evaluate the intestinal barrier function in various studies. These biomarkers can be divided into three main categories. The first category contains substances found in the intestinal epithelial cells which are used to directly reflect the extent of intestinal mucosal damage. There are also some biomarkers that can reveal the damage of intestinal barrier indirectly. Such biomarkers are released into the blood when the intestinal barrier is damaged. Moreover, there are some nonspecific indicators of inflammation that are connected to intestinal mucosal damage. Among the biomarkers listed above, DAO and D-LA have potential clinical application value in the diagnosis of CD activity.

DAO, a key enzyme that catalyzes the oxidation of histamine diamine, is mainly produced in the human small intestine mucosa. The activity of DAO can reflect the functional condition of small intestines [18]. The epithelial cells at the site of damage will release a large amount of DAO. DAO is stably in serum, and therefore, it can reflect the situation of intestinal barrier [19]. D-LA is derived from the metabolism of natural flora within the gastrointestinal tract, and a variety of intraintestinal bacteria can produce D-LA through the glycolysis pathway. The permeability of the intestinal barrier increases when the intestinal barrier is damaged. As a result, D-LA produced by bacteria is released into the blood. Therefore, the serum level of D-LA increases, thereby indicating abnormal intestinal permeability [20]. Mammals do not express D-LA dehydrogenase. Thus, it is reliable to select this biomarker to evaluate intestinal barrier function.

The results of this study showed that the levels of D-LA and DAO in active stage CD patients were higher than those in the remission stage, suggesting that these serological biomarkers can be used to determine whether CD is active or not. This study also found that the serum levels of D-LA and DAO in CD patients in the remission stage were not significantly different from those in healthy population, suggesting that these biomarkers are not appropriate in the diagnosis of CD, but they are only valuable in identifying the active and remission stages of CD. The most common method used to evaluate CD disease activity is the CDAI scoring system. This study found that D-LA and DAO levels in CD patients were strongly correlated with CDAI scores, and the relationship between D-LA and disease activity score was stronger than CRP and ESR, which are currently used in clinical practice. This finding further suggests that detection of D-LA and DAO levels is more effective for evaluating CD activity. According to the ROC curve, D-LA and DAO displayed good diagnostic value when used alone to detect CD activity. Their diagnostic efficacy is not significantly different from that of ESR and CRP. We also calculated the Youden index, and the maximum value was used as the index of accuracy evaluation. When D-LA takes the cut value, the sensitivity and specificity for diagnosing disease activity are 69% and 93%, respectively, which are higher than ESR and CRP. In addition, detection of D-LA and DAO in combination with CRP and ESR significantly improved the diagnostic efficiency, with better sensitivity and specificity.

Accumulating evidence demonstrates that intestinal mucosa healing can reduce clinical recurrence rate, hospitalization rate, and operation rate [21]. Endoscopic score is used to evaluate CD therapy. Twelve patients in this study underwent endoscopy by the same doctor, and Simplified Endoscopic Score for Crohn's Disease (SEC-CD) scores were obtained in strict compliance with the scoring system. However, only 3 patients were in the active stage, and no conclusion with statistical difference can be obtained. Further research is required to address this question.

In clinical practice, disease biomarkers are applied in the control of diseases. Mucosal healing usually reflects the control level of acute inflammatory activity, which indirectly reflects the efficacy of drugs [22]. In this study, we did not evaluate the changes of these biomarkers before and after treatment, but we believe that D-LA and DAO, indicators of intestinal mucosal injury, have a great potential in predicting the efficacy of medical treatment of the disease. Further studies are necessary to validate this finding.

In summary, serum D-LA and DAO possess high diagnostic value for CD activity, and when they are used in combination with the currently used biomarkers, CRP and ESR, their diagnostic accuracy for CD activity was significantly improved. However, the sample size of this study is small. Additional investigations with large sample size are needed to support the results of this study. Moreover, it should be noted that these two indexes have not been compared with the endoscopic scores of CD due to the limitation of sample size. We believe that this study provides valuable information that can help to identify Crohn's disease activity.

## Figures and Tables

**Table 1:** General characteristics of 59 patients with CD.

Characteristics	CD active (*n* = 29)	CD remission (*n* = 30)	*P* value
Age in years, mean (range)	37.2 (20-47)	39.6 (17-55)	0.408
Sex: male, *n* (%)	13 (45)	17 (57)	0.363
Duration of disease in years, median (IQR)	7 (2-11)	7.5 (3-13)	0.504
Smoking history, *n* (%)			0.626
Current	8 (27)	6 (20)	
Former	10 (34)	9 (30)	
Never	11 (38)	15 (50)	
Site of disease, *n* (%)			0.605
Ileal (L1)	2 (7)	1(3)	
Colonic (L2)	13 (45)	17(57)	
Ileocolonic (L3)	14 (48)	12(40)	
Drugs before inclusion, *n* (%)			0.450
Mesalamine	5 (17)	3 (10)	
Azathioprine/6-MP	7 (24)	5 (17)	
Anti-TNF	10 (34)	9 (30)	
Anti-TNF and azathioprine/6-MP	7 (24)	13 (43)	
Number of prior surgeries, mean (range)	0.20 (0-2)	0.28 (0-3)	0.898
Having extraintestinal manifestations, *n* (%)	2 (6)	1 (3)	0.612

CD: Crohn's disease; 6MP: 6-mercaptopurine; IQR: interquartile range. There were no significant differences between groups in the active stage CD patients and remission stage CD patients.

**Table 2:** Comparison of levels of D-LA and DAO among groups.

Group	CD active (*n* = 29)	CD remission (*n* = 30)	*P* value^∗^	Control group (*n* = 28)	*P* value^∗^
D-LA (mg/L)	16.08 ± 4.8	11.16 ± 3.17	<0.001	10.2 ± 3.22	<0.001
DAO (U/L)	11.01 ± 4.49	7.7 ± 3.44	<0.05	6.21 ± 2.34	<0.001

CD: Crohn's disease; D-LA: D-lactate; DAO: diamine oxidase; ^∗^Compared with CD active.

**Table 3:** Accuracy and ROC analyses of D-LA, DAO, ESR, and CRP in differentiating CD active and CD remission.

Diagnostic index	AUC (95% CI)	Standard error	*P* value	Youden (max)	Cut value	Sensitivity	Specificity
D-LA	81.5 (0.692, 0.904)^∗^	0.06	<0.001	0.623	14.91 mg/L	68.97	93.33
DAO	74.8 (0.618, 0.852)^△^	0.07	<0.001	0.525	9.75 U/L	75.86	76.67
ESR	70.5 (0.572, 0.816)	0.07	<0.05	0.386	15 mm/h	58.62	80.00
CRP	78.4 (0.658, 0.881)	0.06	<0.001	0.487	7.85 mg/L	62.07	86.67
Comb	86.1 (0.746, 0.937)^#^	0.05	<0.001	0.659	0.47	75.86	90.00

CRP: C-reactive protein; ESR: erythrocyte sedimentation rate; D-LA: D-lactate; DAO: diamine oxidase; AUC: area under the curve; Comb: combined predictors. Compared with ESR and CRP, ^#^*P* < 0.05; compared with ESR and CRP, ^∗^*P* > 0.05; compared with ESR and CRP, ^△^*P* > 0.05.

## Data Availability

The data used to support the findings of this study are available from the corresponding author upon request.
